# Measurement components of socioeconomic status in health-related studies in Iran

**DOI:** 10.1186/s13104-019-4101-y

**Published:** 2019-01-31

**Authors:** Sediqe Shafiei, Shahram Yazdani, Mohammad-Pooyan Jadidfard, A. Hamid Zafarmand

**Affiliations:** 1 Department of Community Oral Health, Shahid Beheshti University of Medical Sciences, 19839, Tehran, Islamic Republic of Iran; 2 School of Medical Education, Shahid Beheshti University of Medical Sciences, Tehran, Islamic Republic of Iran; 3 Department of Orthodontics, Shahid Beheshti University of Medical Sciences, Tehran, 19839, Islamic Republic of Iran

**Keywords:** Social determinant of health (SDH), Socioeconomic factors, Socioeconomic status (SES), Family characteristics, Household head, Household equipment, Iran

## Abstract

**Objective:**

The *socioeconomic status* (SES) is as a symbol of *social determinants of health* which has a dominant influence on population health. The purpose of this study was *collecting, weighing,* and *determining* the most relevant SES measurement items in Iran.

**Results:**

The SES health studies conducted in Iran was searched from 2007 to 2017. First, the SES items were categorized. Then, each item was weighed based on its *reliability* and *generalizability*. Finally, the necessity of items was *determined*, *weighed*, and *ranked*. This is the *two*-*round Delphi* technique. After weighing 57 SES items, 37 items were selected with ≥ 1 weight and classified in 7 categories. According to the Delphi evaluation, 15 items were identified ≥ 3.5 for measuring SES of Iranian households: *household size*, *head of household education*, *head of household job*, *household monthly income, type of school that children attend*, *house ownership*, *local value of residence*, *number of rooms in the house*, *house area*, *personal computer/laptop*, *smart cell phone*, *3D TV*, *dishwasher, microwave*, and *car ownership*. The SES items for the present society are categorized in 7 domains. The items collected in this study have the most comprehension of all studies related to income, life facilities, and assets.

## Introduction

Our understanding of health and its social determinants has been deepened and is comparable to past decades. Nowadays, *social determinants* are considered as the key factors of health quality and play an important role in the macro concept of health. These factors along with health services *directly* or *indirectly* can influence the health status of people in communities.

Each of the social determinants of health such as *income*, *education*, *occupation*, *nutrition*, and the *social class* have a much larger role than biological factors in human health.

The socioeconomic status (SES) is the most influential determinant of health [1, 2]. SES is a complex and multidimensional construct, which requires a standardized format of measurement for each community. That is a concept that is used not only to measure *social components of health* but also to measure *socioeconomic inequalities of health* [3]. Generally, SES is defined as the position of an individual or of a household within a society. It is a combination of occupation, education, income, wealth, and residence neighborhood [4, 5]. Given the above-mentioned issues, building of an appropriate tool for measuring SES can be a significant contribution for *planning* and *policy*-*making* in health system, both at micro & macro levels [6].

In developing countries, the SES survey is a challenging issue in data collection for assessing and monitoring health equity. Therefore, researchers have concluded that development of a structured format for each society is necessary for SES measurement [7]. Reviewing studies performed in Iran and additionally based upon a systematic review conducted by Mahdavian et al. [8], there is a tremendous discrepancies in measuring SES method.

Due to such diversities and given the important role of SES in health studies, there is a need for a unified tool to collect socioeconomic data for an each community based on its specific circumstances and its level of technology development. Plus, the SES measurement tools are dynamic, i.e. an item that can be a household SES indicator for a population at a period of time may not be applicable later on. In other word, ever-changing quality of life standards may discredit an SES indicator that was valid an earlier time.

This study has tried to collect and edit a set of *most appropriate items* that could well represent the SES criteria for the Iranian society. These items can be used for development of SES measurement as a tool applicable for related researches in different dimensions. Furthermore, it can provide a *unified platform* to compare the results of different studies. In addition, this study *gathered*, *weighed*, and *determined* the *necessity of items* for measuring the SES for Iranian society.

## Main text

### Materials and methods

The mixed method of *review study* and *Delphi method* was applied. A critical review was carried out to collect items used for measuring SES in Iran. The items were weighed based upon the validity and generalizability of the extracted item. The weighed items were ranked based upon the experts’ opinion.

### Search review strategy

A literature review was performed in *PubMed* database covering during 2007–2017 using the following keywords: *SES, socioeconomic factors, wealth, welfare, asset, tool, develop, instrument, measure,* and *Iran*. Appropriate operatories (AND, OR, NOT) and appropriate filters were used to focus the search goals. A search for articles published in Farsi was performed using of the above-mentioned keywords in *Google Search Engine*.

### Selection process

Full text of articles was reviewed in the health field that looked on SES items. *Backward search* was also considered for original questionnaire.

The extracted SES measurement was entered in a table and categorized in 7 socioeconomic domains, such as: (a) *demographics*, (b) *purchasing ability*, (c) *employment status*, (d) *literacy/education*, (e) *housing and accommodation status*, (f) *home appliances*, and (g) *personal assets*. By designing an Excel spreadsheet, the frequency of each SES item was presented for an individual paper.

### The scoring method

Then, to develop the most valid list of SES items related to the community of Iran, articles were weighed based upon two criteria: (1) the validation of study method, and (2) generalizability of SES items at the nationwide scale. The scoring method was based upon the consensus of experts’ panel. Two review authors (SSh, ShY) independently assessed the scoring approach in the present study, with any disagreements resolved by discussion and consensus of the team. The scores allocated to each article for validation were determined arbitrarily by the research team prior to the assessment, according to the American Psychological Association (APA) guideline [9]. The scoring system was as follows:

I. *1 point*: The *strong statistical method of Principal Factor Analysis* (PFA) or *Principal Component Analysis* (PCA) was used to validate the SES items.
II. *0.6 point*: Validation of the SES items was evaluated using an *experts’ panel*.
III. *0.5 point*: If the article cited *another article* with an appropriate validation method.
IV. *0.3 point*: When only *internal consistency* of items was assessed.
V. *0.1 point*: When the validation technique was *not clear*.

Based upon the experts’ consensus, the scoring system for generalizability of an article was as follows:

I. the studies that were conducted in *less than half of the provinces* of Iran, scored as *0.25 point*.
II. the studies that were conducted in *more than half of the provinces* of Iran, scored as *1 point*.

### Determining the weight of each SES items

The weight of each study was calculated through multiplication of *validation score* by *generalizability score*. The weighed items were added to the excel table. Then, weighing of each item calculated by sum of the scores recorded for each article in the excel table. Next, the items that were weighed higher than score of one—assuming that there was at least one validated article about it—were selected to get experts opinion.

### Determining the final SES items

In order to determine the final SES items, the two-round Delphi method was used to obtain structured experts’ opinions, based upon the five-point Likert scale. A comment section was also provided for further explanations. After receiving the first round of Delphi method, the percentage of Likert scale was calculated according to expert responses for each SES items. Afterward, the unique filled questionnaire which contained the percentage of experts responded to each Likert score with their comments were returned for the second round of evaluation. It is of statistically significance to provide each individual expert opinion visible for all other experts. This has the advantage that experts can freely revise their first round opinions in the second round of evaluation, as well, increasing the dependable face validity.

### Selection of experts’ panel

Initially, 15 experts for Delphi method were selected *using* purposive *and* snowball sampling *techniques based* on their experience in the related subject. Finally, 11 experts accepted to participate in the study. The composition of the group was from a wide range of academic affiliations: 4 public health specialists, 2 health economists, 1 health policy specialist, 2 healthcare managers, and 2 socialists.

## Results

After reviewing the titles, abstracts and full-text of the articles, 60 related articles were selected. It contained 45 English articles from PubMed database and 15 Farsi articles from the Google search engine. Items that were used to measure SES in selected articles are listed in Table 1.

**Table 1:** Items that were used to measure SES in selected articles

First author	Year	Geographical	Target group	Validation	Language	SES items
Doulabi [11]	2017	Tehran	1036 children 36–60 months	+	English (E)	Parent’s education, house ownership, floor area of the housing unit, having one or two cars, monthly income, computer, number of family members
Khajavi [13]	2017	31 provinces in Iran	–	+	E	Home area, number of rooms, car, television, refrigerator, oven, vacuum cleaner, washing machines, media players, cell phone, telephone, bathroom, kitchen, gas pipe line
Almasi-Hashiani [14]	2017	Tehran	5170 women	+	E	Vacuum cleaner, handicraft carpet, laptop, freezer, dish washing machines, private cars, touch mobile, three-dimensional TV, side-by-side refrigerator, microwaves, number of rooms and area of residence
Kelishadi [15]	2017	30 provinces in Iran	23,183 school students	+	E	Parental education, parents’ job, possessing private car, school type (public/private), and having personal computer in home
Kia [16]	2017	31 provinces in Iran	29,609 household	−	E	TV sets, refrigerators, freezers, radios, cell phones, wristwatches, computers, laptops, microwaves, washing machines, vacuum cleaners, dish washing machines, cars, heating and cooling systems, fuel in the kitchen, access to internet, sources of drinking water, bathrooms, number of rooms, toilets, home ownership
Mosallanezhad [17]	2017	Tehran	75 yeas	−	E	Overall years of education, job status and monthly family income
Maharloue [18]	2017	Shiraz	3400 households	+	E	Education level and occupation head of household and partners, household income
Ayubi [19]	2017	Zanjan city	1064 student high schools	+	E	Car, washing machine, dishwasher, fridge/freezer, vacuum cleaner, personal computer and laptop, microwave, LCD or LED TV
Tajik [20]	2016	Falavarjan	302 patients	+	Persian (P)	Type of home, home area, number of rooms, personal car, motorcycle, furniture microwave, washing machine, dishwasher, TV, freezer, vacuum cleaner, cell phone, landline, cooler type, kitchen space
Kavefirooz [21]	2016	Tehran	384 women	−	P	Education, family income, occupation, place of residence, type of home, parental education
Mostafavi [22]	2016	16 provinces in Iran	2494 subjects 10–18 years	+	E	Personal home, car, computer, school type (private/public)
Mirmoghtadaee [12]	2016	30 provinces in Iran	13,486 students	+	E	House, car, computer, parental education and occupation, school type (private/public)
Safiri [23]	2016	30 provinces in Iran	13,486 student 6–18 years	+	E	Parents’ education, parents’ job, private car, school type (public/private), type of home (private/rented), and having personal computer
Ahmadi [24]	2016	Golestan Province	50,045 40–75 years	−	E	Family asset, ethnicity, sex, employment status, age at starting the first job, size and the status of house
Alhossaini [25]	2016	Isfahan Najafabad Arak	10,745 people aged ≥ 19 years	−	E	Ownership of a house, car, personal computer, health insurance support
Heshmat [26]	2016	31 provinces of Iran	14,136 aged ≥ 15 years	−	E	House ownership, number of rooms, TV, cell phone, car, freezer, washing machine, dish washing machine, phone, microwave, access to internet, occupation and education heads of the families, number of family members
Rezazadeh [10]	2016	Urmia	723 participants aged 20–64	−	E	Tap water, gas, electricity, telephone, bathroom, toilet, color TV, black and white TV/stove with oven or without oven, refrigerator, freezer, vacuum cleaner, washing machine, motorcycle, car, number of cars, mobile phone, number of mobile phones, computer/laptop, internet connection
Tavakoli [27]	2016	Tehran	292 women 60 years	−	E	Home ownership, monthly income and the number of essential item for living
Pasdar [28]	2015	Kermanshah	687 women 65–25 years	−	P	Occupation, education, income
Keshtkar [29]	2015	Arak and Sanandaj	2617 people ≥ 20 years	+	P	Education, housing ownership, home area, mobile phone, freezer, washing machine, dishwasher, computer, internet access, car
Naghibi [30]	2015	Mazandaran	184 children under 5-year	−	P	Parental education, parent’s occupation, place of residence, housing ownership, family income
Roudsari [31]	2015	Tehran	722 people 30–64 years	−	E	Age, gender, occupation status, education, duration of residence in Tehran, ethnicity, religion, marital status, number of children
Abobakri [32]	2015	East Azerbaijan	700 households	+	E	Value of housing, health expenditure of household, occupation rank, income, education of head of household, value of personal car
Bahramian [33]	2015	Tehran	20,320 adult 15–64 years	+	E	Average living area per person, room capitation per person, landline, mobile phone, bathroom, kitchen, toilet, car, motorcycle, refrigerator, microwave, oven, computer, dishwasher
Ramezani Doroh [34]	2015	Shiraz	852 men 716 women	−	E	Monthly income
Ghorbani [35]	2015	Tehran	1100 adult	+	E	Education, house area per capita, house value based on location, house ownership, having a car, computer, dishwasher, steam-cleaner, microwave, internet access
Baygi [36]	2015	27 provinces of Iran	5682 students 10–18 years	−	E	literacy, family permanent income (family assets), employment rate
Morowatisharifabad [37]	2015	Ardakan and Yazd	188 children 3–5 years	+	E	Parents’ education and occupation, the size of the house (m^2^), whether the house had a yard, and if so, could it be used as a playground by the child
Mashayekhi-Ghoyonlo [38]	2015	Mashhad	140 patients	+	E	Level of education, job, income, monthly savings, place of residence, home ownership, car ownership
Najafianzadeh [39]	2015	Arak	373 rural households	+	P	Parent education, income, sofa, handmade carpet, freezer, refrigerator, washing machine, dishwasher, microwave, computer, car, personal home
Shishehgar [40]	2014	Tehran	210 pregnant women	+	E	Marital status, occupation and education level, monthly income, place of residence, number of people per household, cost per square meter of their house, car, computer
Cheraghian [41]	2014	Tehran	69,173 25–64 years	+	E	Owning fridge, personal computer, telephone, mobile phone, washing machine, microwave oven, car, motorcycle, kitchen, bathroom, toilet, house ownership, number of rooms per capita, area of the house
Tajik [42]	2014	28 provinces of Iran	27,000 households	+	E	Kitchen, bathroom, vacuum cleaner, washing machine, freezer, personal computer
Mokhayeri [43]	2014	Tehran	–	−	E	Job
Eslami [44]	2014	Tehran	700 adult 18–64 years	−	E	Age, gender, marital status, having children, educational level, employment, profession, annual income, perceived financial strain
Kavosi [45]	2014	Shiraz	100 patients	+	E	Age, sex, education, occupation, insurance
Mohebbi [46]	2014	Tehran	499 individuals 20–50 year	−	E	Educational level, family income, house ownership, household size and number of persons
Heydari [47]	2014	Ahvaz	350 students university	−	E	Father’s and mother`s education level, father’s career, family’s income, relative price of own dwelling, purchasing power for buying a dwelling
Eslami [48]	2014	Mashhad	359 citizens	−	P	Income, economic class, housing ownership, education
Pasdar [49]	2014	Kermanshah	500 households	−	P	Maternal income, mother’s education, father’s education, mother’s occupation, father’s occupation
Ghodratnama [50]	2013	Ahvaz	370 students university	+	P	Income—economic class—housing ownership—parental education
Nejhad [51]	2013	28 provinces of Iran	3472 patients	+	E	Phone at home, cell phone, washing machine, dish washer, microwave, camcorder, car, residential Area, number of rooms, main cooling devices, main cooking device
Naghibi Sistani [52]	2013	Tehran	1031 18–65 years	−	E	Living area in square meters per person, education, employment
Nazari [53]	2013	30 provinces in Iran	58,421,420 Iranian ≥ 10 years	−	E	Number of family, % of individuals literate in family; % of individuals with employment in family, % of family members are students, car, access to the Internet, accommodation size, numbers of rooms, kitchen, gas pipe lines, house ownership, motorcycle, bathroom, effluent system, existence of disabled individual in the family.
Khayatzadeh [54]	2013	Tehran	220 mothers	−	E	Educational level (education of mothers), occupational status (both parents), income (both parents) and housing situation (the type, size of housing and the total number of rooms)
Asefzadeh [55]	2013	Qazvin	878 persons	−	P	Education, job classification, income, income percentile
Nedjat [56]	2012	Tehran	2464 residents of Tehran	+	E	Number of rooms and living area per capita, separate kitchen, bathroom, computer, washing machine, freezer, dishwasher, vacuum cleaner, personal car, mobile phone, color TV, video or DVD player, telephone
Fakhri [57]	2012	Mazandaran province	698 students	−	E	Occupation of the father of the family
Morasae [58]	2012	Tehran	22,135 people ≥ 15 years	+	E	Personal computer, freezer, car, motorcycle, mobile phone, kitchen, bathroom, landline, toilet, house ownership, residence area per capita, number of rooms per capita
Zolala [59]	2012	28 provinces of Iran	Ecological study	−	E	Unemployment, urbanization and literacy in the different provinces
Rohani-Rasaf [60]	2012	Tehran	Ecological study	+	E	House ownership, room per person, area per capita, having bath, kitchen, toilet, car, phone, cell phone, freezer, computer, years of education
Payab [61]	2012	Ray city	430 mothers	−	P	Level of education, job position of head of household and mother, housing ownership, sofa, handmade carpet, refrigerator freezer, washing machine, dishwasher, microwave, computer, car
Yaghoubi and Enayat [62]	2012	Ahvaz	384 students 18–14	+	P	Maternal occupation, maternal income, maternal education
Donyavi [63]	2011	Tehran	1283 patients	−	E	Living area in square meters per person, education, employment
Fazeli [64]	2010	North-East of Iran	86 patient	−	E	Clean and tidy appearance, level of literacy, having a known professional career, unemployment, longer than 3 months in Iran, good job, monthly income, place of living
Sheykhmounesi [65]	2010	Sari	40 adults 40 children	−	P	Housing ownership, education level, family size, occupation, secondary occupation, job wife, car, mobile phone, agricultural land, insurance type
Garmaroudi [66]	2010	Tehran	1000 households	+	P	Household education, wife education, home area, home prices, car, computer
Montazeri [67]	2008	Tehran	4163 ≥ 15 years	−	E	Years of formal education
Ansari [68]	2008	Zahedan	240 university students	+	P	Father’s education, mother’s education father’s job, mother’s job, income
Hosseinpoor [69]	2007	29 provinces of Iran	524,111 households	+	E	Number of rooms per capita, car, motorcycle, bicycle, fridge, TV, telephone kind of heating device

After removing duplications, 57 items were categorized in seven domains: (I) *Demographic*, (II) *Purchasing ability*, (III) *Education*, (IV) *Employment*, (V) *Housing status*, (VI) *Home appliances*, and (VII) *Personal assets* (Table 2). After weighing the mentioned items, the above items were decreased to 37, if they received one or more point weight (≥ 1).

**Table 2:** These present 37 items are product of the second step of the study that gained the score of ≥ 1

Item name	Frequency	Weight^a^	Median^b^
*Demography*			
Ethnicity, religion age, gender marital status	5	0.65	–
Household size	9	3.45	4.5
Urban residency	2	0.05	–
Existence of disabled in the family	1	0.5	–
*Purchasing* *power*			
Monthly income	24	3.975	5
Monthly saving	1	0.75	–
Health expenditure	1	0.15	–
Annual income	1	0.025	–
Purchasing power	2	0.05	–
Insurance	3	0.225	–
*Literacy*			
Head of household education	39	9.8	5
% of literate individuals in family	1	0.5	–
% of family members who are students	1	0.5	–
School type (public/private)	4	3.5	4
*Employment status*			
Head of household job	33	7.35	5
Second job	1	0.025	–
% of individuals with employment in family	1	0.5	–
Unemployment	1	0.125	–
*Housing and accommodation status*			
Whether the house had a yard	1	0.15	–
Type of home	13	6.875	4.5
Number of rooms	25	7.725	4
Main cooling devices	3	1.75	3
Home area	17	4.55	4
Gas pipe lines	4	2.125	2
Landline	11	5.125	1
Toilet	6	1.625	1
Internet access	6	2.125	3
Source of water	2	0.625	–
Electricity	1	0.125	–
Bathroom	10	4.375	1.5
Kitchen	9	4	1
Effluent disposal system	1	0.5	–
Kind of heating device	2	1.5	3
House value based on location	9	4.075	4
*Home appliances*			
Vacuum cleaner	8	4.375	2
Washing machine	13	5.75	3
Dish washing machine	12	4.875	4
Media player	2	1.25	2
Hand carpet	4	1.525	3
Main cooking device	2	1.125	2
Microwave	10	4.625	4
Steam-cleaner	1	0.25	–
Furniture	4	0.775	–
Camcorder	1	1	2.5
Radio	1	0.5	–
3 dimensional TV (LCD, LED)	2	1.25	4
Color TV	8	3.775	2
Refrigerator	10	3.9	2
Side-by-side refrigerator	1	1	3
Freezer	13	5	2.5
Oven	3	1.5	2
*Personal asset*			
Ownership of car	30	13.625	4
Motorcycle	7	2.625	2
Bicycle	1	1	2
Mobile	13	4.9	3
Smart phone	1	1	3.5
Personal computer/laptop	22	10.125	3.5

The final median score was set at the level of ≥ 3.5, arbitrarily

^a^The weight of each study was calculated from: validation score × generalizability score

^b^Median score of appropriate rates were reported for items included in the second of the Delphi method

Among 37 items, the ones that gained the score higher than the median of 3.5 based upon the consensus within the experts’ panel opinions, were set as the basis of selection. That concluded with 15 items suitable for SES measurement. These items included: (1) *household size*, (2) *head of household education*, (3) *head of household occupation*, (4) *household monthly income*, (5) t*ype of school that children attend (public/private)*, (6) *House ownership*, (7) *Local value of residence*, (8) *Number of rooms in the house*, (9) *House area*, (10) *Personal computer/laptop*, (11) *Smart cell phone*, (12) *Three*-*dimensional television*, (13) *Dishwasher*, (14) *Microwave*, and (15) *Car ownership*.

## Discussion

The items of this study were related to indirect indicators and assessed beside the direct indicators of assets. While reviewing the literature, three studies were only found used a close approach to the present study method. Abubakri et al. [32] developed and validated a questionnaire for assessing SES in urban households for health studies. They chose SES items from international literature and used the expert panel’s opinions for adjustment. In their questionnaire, personal vehicle was the only item scaling the asset. However, the present study selected the SES items from national studies performed for Iranian community. Further, some indicators included in this investigation have a special emphasis on many other asset indicators.

Another study concluded that 6 items out of 33 items of the household cost-income questionnaire, established by the Center for National Statistics of Iran (CNSI), were sufficient for measuring the SES of Iranian households [42]. These items comprise of: kitchen, bathroom, vacuum cleaner, washing machine, freezer, and personal computer. In fact, this study considered asset items much limited to those of the CNSI’s questionnaire and no gold standard was used to compare the results of regression analysis.

In a questionnaire designed by Garmaroudy et al. [66] six items were used to identify SES items of householders in Tehran, including: head of households and his spouse education, area and price of house, personal vehicle, and computer set. Of these items, only two items were directly related to assets, and two-thirds of the total weight of measurement tool was allocated to education. Needless to mention that education has less quantification value for SES evaluation.

In the above-mentioned studies, with the exception of one study [42], the SES measurement tools have been developed and validated by focusing on either a specific subgroup of population or an international community, not for a nationwide model. Therefore, their results cannot be generalized to all Iranian households. On the contrary, this study has pointed out the comprehensive items that *not only* structured for this public *but also* can be applied to nationwide Iranian households.

**Fig. 1:**
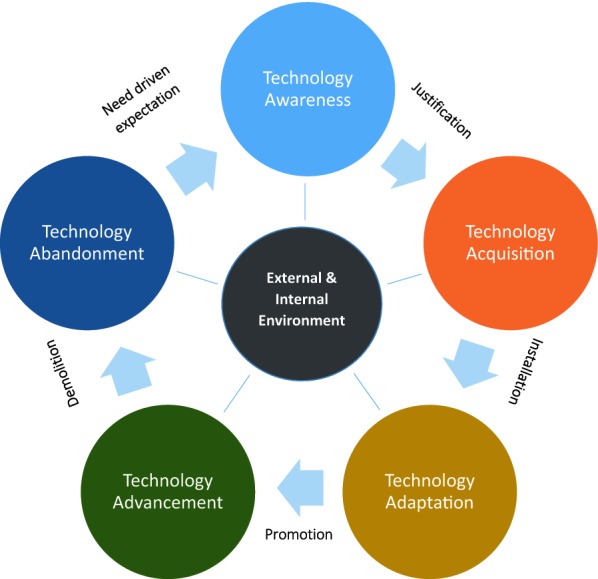
Technology cycle time comprises of: Technology Awareness, Technology Acquisition, Technology Adaptation, Technology Advancement, Technology Abandonment. This would define the internal and external environment

It is of significance to mention that SES is composed of different dimensions and domains that may change or lose their validity over time. However, this fluctuation is not similar for all defined items. In other word, there are items that are more dependent to technology and are consequently subjected to change their creditability, accordingly. For example, based upon a study that conducted at one time cell phone was a luxury device and in a later time becomes a standard life accessory. Another example is the internet accessibility which is rapidly expanded for public use during the past decade. This is socially recognized as *technology acquisition* and *technology advancement* [70] (Fig. 1). This highlights the need for renewing the SES measurement tools, including the combination of items used in the tool, at appropriate time intervals. This issue is applicable to the findings of this study in the future, as well.

To develop a more precise measurement tool, there are *a few points* that should be taken into consideration. *First*, the items about area of the house, household income or the number of rooms in the house should be adjusted based on the number of household members. *Second*, due to the nature of some jobs in the community, such as day-labor or farmer, it is preferable to refer the annual income rather than monthly income. Also, the number of jobs that people may be occupied with should not be neglected. *Third*, the concept of residence should not be limited to a rental home since the ownership of other residentials, commercial unit, or a vacational residence. *Fourth*, the regional-value of residential location should be concomitantly considered with the house area. Therefore, the house price and rent can be one of the functions of the economic value of the residence area. *Fifth*, assets such as: vehicles, laptop, smartphone, 3D TV, dishwasher and microwave fall into different price categories due to their various features and brands. As such, the price of a selected utensil may place from a very low to very high range of a price. Thus, structuring an inclusive SES questionnaire requires more in depth queries.

## Conclusions

This study comprehended fifteen items were collected in this study in 7 domains for SES criteria as a dependable measurement tool for Iranian households. Obviously, as the technology changes over time, the SES measurement tools are required to be revised. The methodology used in this study provides an on-going basis for updating the SES tools.

## Limitation of the study

The present study faced with some limitations for implementation. First, there was limited number of articles structured with a well-designed study for SES evaluation. Second, the limited number of publications in our national level did restrict the authors for designing a solid study.
